# Development and Fabrication of Biocompatible Ti-Based Bulk Metallic Glass Matrix Composites for Additive Manufacturing

**DOI:** 10.3390/ma16175935

**Published:** 2023-08-30

**Authors:** Po-Sung Chen, Pei-Hua Tsai, Tsung-Hsiung Li, Jason Shian-Ching Jang, Jacob Chih-Ching Huang, Che-Hsin Lin, Cheng-Tang Pan, Hsuan-Kai Lin

**Affiliations:** 1 Institute of Materials Science and Engineering, National Central University, Taoyuan 32001, Taiwan; 2 Department of Mechanical Engineering, National Central University, Taoyuan 32001, Taiwan; 3 Department of Materials and Optoelectronic Materials, National Sun Yat-Sen University, Kaohsiung 80424, Taiwan; 4 Department of Mechanical and Electro-Mechanical Engineering, National Sun Yat-Sen University, Kaohsiung 80424, Taiwan; 5 Department of Materials Engineering, National Pingtung University of Science and Technology, Pingtung 91201, Taiwan

**Keywords:** metallic glass (MG), biocompatible, selective laser melting (SLM), additive manufacturing (AM)

## Abstract

Ti-based metallic glasses have a high potential for implant applications. The feasibility of a new biocompatible Ti-based bulk metallic glass composite for selective laser melting (SLM) had been examined. Therefore, it is necessary to design a high-glass-forming-ability Ti-based metallic glass (∆T*_x_* = 81 K, γ = 0.427, γ_m_ = 0.763), to fabricate a partial glass-formable spherical powder (the volume fraction of the amorphous phase in the atomized Ti-based powders being 73% [size < 25 μm], 61% [25–37 μm], and 50% [37–44 μm]), and establish an SLM parameter (a scan rate of 600 mm/s, a power of 120 W, and an overlap of 10%). The Ti_42_Zr_35_Si_5_Co_12.5_Sn_2.5_Ta_3_ bulk metallic glass composite was successfully fabricated through SLM. This study demonstrates that the TiZrSiCoSnTa system constitutes a promising basis for the additive manufacturing process in terms of preparing biocompatible metallic glass composites into complicated graded foam shapes.

## 1. Introduction

Selective laser melting (SLM) is a promising additive manufacturing (AM) technique. SLM involves the direct fabrication of three-dimensional (3D) metallic parts with complex structures and a high density (higher than 99%) by a high-power density laser beam that melts metallic powders in a vacuum or inert-gas protection system through a computer-aided design (CAD) model [1,2,3,4]. The components are fabricated by selective melting and fusing powders within and between the layers. The porous structure and ready-to-use parts are easy to produce. Compared with conventional manufacturing techniques, SLM has a fast cooling rate (10^3^–10^5^ K/s) during the solidification process [5]. This allows the bulk materials to form very fine nonequilibrium microstructures and improves their mechanical properties [6,7,8].

SLM can be beneficial in medicine. Its greatest advantage is that it enables one to freely customize and personalize medical products, which can reduce patient recovery time and improve surgical outcomes [9,10]. The ideal bionic metallic implant should possess biocompatibility, a porous structure, osteoconductivity or osteoinductivity, and a suitable strength and stiffness [11]. Conventional implants are usually in a solid block form; they do not have the aforementioned characteristics that SLM does. SLM further offers the advantage of a high cost effectiveness. SLM is more cost-competitive for small-volume, large-variety productions, such as spinal, dental, and bone productions, each of which has a highly complex shape [9]. The fast fabrication of SLM allows a medical product to be made within several hours, leaving enough time for clinicians to respond to contingencies [12,13,14]. These characteristics of SLM are difficult to obtain through traditional manufacturing methods.

Metallic materials are often the optimal choice for biomedical implants, especially for hard tissue replacements [15,16,17], because of their high tensile strength, fracture toughness, and greater suitability for load-bearing than ceramics or polymeric materials [18,19,20,21]. Among metallic alloys, titanium and its alloys (e.g., Ti-6Al-4V and Ti-6Al-7Nb) are regarded as the preferred materials and are mostly used for biomedical implants in the fields of trauma and orthopedic surgery [21,22]. However, they still create several problems regarding long-term tribological behavior and have raised some health concerns [23,24]. The major problems for the Ti-6Al-4V implant are the mismatch of the Young’s modulus (E) with human bone (human bone: E = 10–30 GPa; Ti-6Al-4V: E = 110–120 GPa) and the release of toxic metallic ions and particles through corrosion or wear processes. The large difference of E causes nonuniform loading of the bone interface in implants, resulting in stress-shielding effects. This increases patient recovery time and changes the bone shape [19,20]. In addition, toxic factors might lead to oversensitivity, immune rejection, and tissue loss. Therefore, researchers have been developing new titanium alloys with suitable mechanical properties and a good biocompatibility for bone implants [25,26,27,28,29,30,31,32,33].

In the past two decades, a new metallic material category, the amorphous alloy or metallic glass (MG), has attracted research attention for its potential biomedical applications [34,35,36,37,38]. Due to their inherently amorphous properties, these MGs do not have crystal structural defects, such as dislocations, twins, or grain boundaries. MGs also possess a homogenous chemical constitution with a higher hardness, higher strength, lower Young’s modulus, larger elastic strain, and much better corrosion resistance compared with their counterpart, crystalline alloys [39,40,41,42,43]. Developments in Ti-based MGs without a toxic element, Ti_60_Zr_10_Ta_15_Si_15_, Ti_60_Nb_15_Zr_10_Si_15_, and Ti_42_Zr_40_Ta_3_Si_15,_ have been found to be superior to crystalline Ti-alloy counterparts from a medical-implant point of view [33,34,39,42]. More recently, studies have reported that a new type of bulk metallic glass foam (BMGF) with the ability to support cell growth [35,36,42] can further reduce the effects of stress shielding and problems with toxic elements. However, through the space holder powder metallurgy method, the BMGFs are able to provide only one fixed porosity in one product, thus still facing limits in their application [31]. On the other hand, the Young’s modulus of the Ti-based BMG is around 80 GPa, which is still higher than the Young’s modulus of 15–25 GPa in the longitudinal direction of cortical bone [44]. These cannot completely satisfy the requirements of human bone with respect to cellular activity and load bearing. The best solution is to use an AM process by an SLM method to directly fabricate high bionic-gradient porous-implant Ti-based MGs on a CAD data model.

In this study, we attempted to improve the glass-forming ability (GFA) of a Ti_42_Zr_40_Ta_3_Si_15_ system by adding biofriendly Sn and Co elements. The starting Ti-Zr-Ta-Si alloy has been extensively studied in our laboratory for its in vitro and in vivo biocompatibility [39,42]. This starting alloy is well suited as a metallic biomaterial. The alloy is free of toxic elements Ni and Cu and of the expensive element Pd. Gas atomization was used to prepare fully or partially glass-formable spherical powder with a shape factor of 0.85–1 and a particle size distribution of d_50_ ≤ 45 μm for the SLM process. The fabrication of Ti-based bulk metallic glass composite (BMGC) through SLM was analyzed.

## 2. Experimental Procedures

Ingots with a nominal composition of Ti_42_Zr_w_Ta_3_Si_x_Sn_y_Co_z_ (w + x + y + z = 55 at %) were prepared by arc melting under a Ti-gettered argon atmosphere. The purity of the raw element was above 99.9%. The ingots were turned over and remelted at least four times to ensure their chemical homogeneity. Ribbon samples were prepared by subjecting the ingot to induction melting and ejecting it onto a water-cooled copper wheel (with a tangent speed of 25 m/s and a gap of 0.2 mm between the quartz nozzle and the wheel surface) in an argon atmosphere. The microstructure of all ribbons was examined by X-ray diffraction (XRD; D8A, Bruker, Billerica, MA, USA) with monochromatic Cu-Kα radiation. The thermal characteristics (glass transition temperature, T*_g_*, and crystallization temperature, T*_x_*) were measured at a heating rate of 40 K/min with differential scanning calorimetry (DSC; DSC 1, Mettler Toledo, Greifensee, Canton of Zürich, Switzerland). The melting behavior (characterized by the melting point, T_m_, and liquidus temperature, T*_l_*) was measured at a heating rate of 20 K/min using high-temperature DSC (HT-DSC; DSC 404 F3, Netsch, Selb, Bavaria, Germany).

The fully or partially glass-formable powders were fabricated by using self-designed gas atomization equipment on the south campus of the Industrial Technology Research Institute, Taiwan. Metal powders obtained by gas atomization have a perfectly spherical shape and a high cleanliness level. The particle size distribution of as-prepared alloy powders was analyzed using a Dry-Type Laser Particle Size Analyzer (Mastersizer 2000, Malvern instruments, Malvern, Worcestershire, United Kingdom). The microstructures of each size-ranged alloy powder were examined using XRD analysis and transmission electron microscopy (TEM; JEM-2100, Joel, Akishima, Tokyo, Japan; operated at 200 kV). The morphology and chemical composition of alloy powders were examined using scanning electron microscopy (SEM; F50 Inspect, FEI, Hillsboro, OR, USA; operated at 25 kV) equipped with an electron dispersive spectrometer.

The gas-atomized powders with a particle size of 15–45 µm were sieved and divided for the SLM process. Specimens with dimensions of 10 mm × 10 mm × 3 mm were produced using SLM in an argon environment with an AM system (LUMEX Avance-25, Higashimorida, Fukui, Japan). Several parameters, including the laser power and the scan speed, were employed with a layer thickness of 50 μm. A scan strategy with a bidirectional scanning vector and 90° rotation between consecutive layers was applied. The effect of the SLM process on the microstructure of the Ti-based bulk metallic glass was investigated using XRD and TEM.

## 3. Results and Discussion

### 3.1. High Glass-Forming Ability for Ti-Based Metallic Glass

Several empirical and theoretical criteria for an easy MG formation were indicated by the findings. These criteria deal with electronic, topological, compositional, kinetic, and thermodynamic conditions that facilitate the formation of a short-range-order structure. They include Turnbull’s deep eutectic rule and Egami’s atomic size rule [45,46]. Based on the deep eutectic rule, the alloys with lower T*_l_* would inherently have a higher GFA because the nucleation and growth of the crystalline phase is directly related to the cooling temperature and time. Alloys with lower T*_l_* temperatures are in a liquid state at lower temperatures, making the rapidly cooled melt’s transformation into a crystalline phase more difficult. Therefore, an alloy composition at or near the deep eutectic point becomes easier to transform into the glassy phase before forming the crystalline seed [47]. Using the physical concept of strong binary eutectic clusters as a basis, Lu et al. [48,49] proposed the following equation to identify the deep eutectic composition:

C_am_ = α (Eu_A-B1_) + β (Eu_A-B2_) (1)

**Figure 1:**
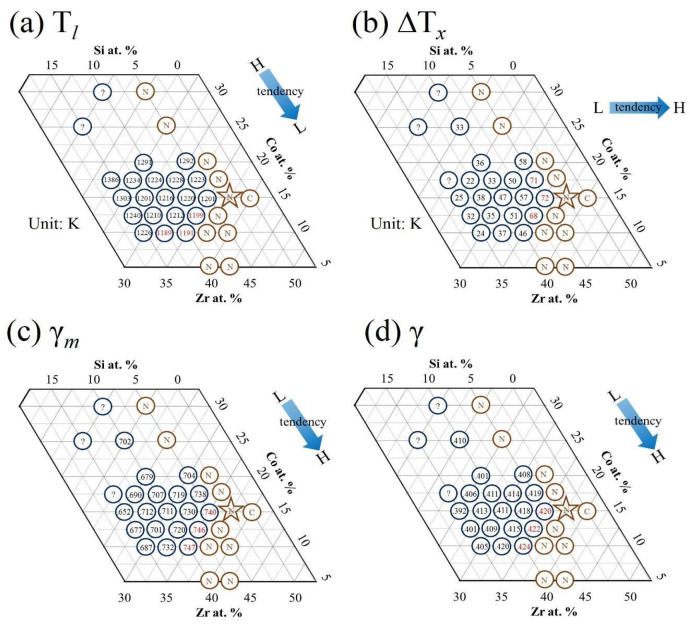
Composition diagram for Ti_42_Ta_3_-Zr-Co-Si alloy ribbon. A star symbol indicates predicted composition using the calculation of binary eutectic clusters. An *N* indicates that the XRD pattern of ribbon exhibited a broad diffuse peak with a nanocrystalline peak. A *C* indicates a very sharp crystalline peak. The other compositions are of the MG structure. (**a**) Liquidus temperature, T*_l_*, (**b**) supercooled liquid region, ∆T*_x_*, (**c**) GFA index, γ_m_, and (**d**) GFA index, γ.

**Figure 2:**
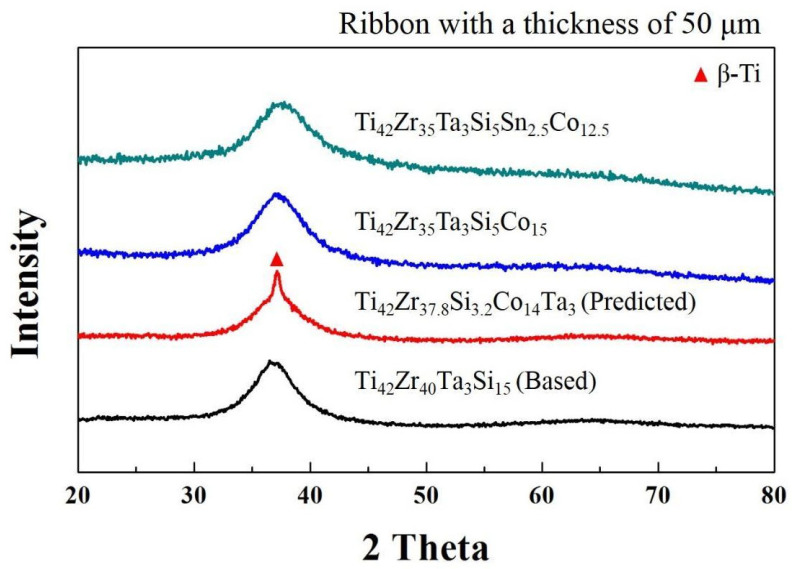
XRD patterns of Ti–Zr–Ta–Si–Sn–Co melt-spun ribbons.

αΔH_A-B1_ = βΔH_A-B2_, (2)

where C_am_ indicates a composition range bounded by the two deep binary eutectic compositions, Eu_A-B_ is the eutectic point for the binary cluster A-B, ΔH_A-B_ is the mixing heat for the binary cluster A-B, α and β are constants, and α + β = 1. Ti, Zr, Co, and Si can form the binary deep eutectics Ti_77_Co_23_, Ti_86_Si_14_, Zr_91.2_Si_8.8_, and Zr_78.5_Co_21.5_ [50]. The mixing heats for the atom pairs of Ti–Co, Ti–Si, Zr–Si, and Zr–Co are −28, −66, −84, and −41 kJ/mole, respectively [51]. Following Equations (1) and (2), the possible eutectic compositions (C_am_) in the Ti_42_Ta_3_(Zr–Si–Co) system can be calculated, and they yield a composition of Ti_42_Zr_37.8_Si_3.2_Co_14_Ta_3_. Figure 1 presents the GFA factors of the melt-spun Ti_42_Ta_3_(Zr–Si–Co) ribbons, which included the liquidus temperature (T*_l_*) (Figure 1a), supercooled liquid region (∆T*_x_* = T*_x_* − T*_g_*) (Figure 1b), GFA index [γ_m_ = (2T*_x_* − T*_g_*)/T*_l_*)] (Figure 1c), and GFA index [γ = T*_x_/*(T*_g_* + T*_l_*)] (Figure 1d). Figure 2 presents the XRD pattern of the melt-spun Ti_42_Ta_3_(Zr–Si–Co) ribbons with various compositions. As evidenced in Figure 1 and Figure 2, the XRD pattern of the predicted composition exhibited a broad diffuse peak with a nanocrystalline peak, implying that some physical properties were neglected. We attempted to measure various parameters of the predicted alloy near the eutectic compositions. The true eutectic point clearly shifted into a lower Zr and Co but higher Si content (i.e., Ti_42_Zr_32.5_Si_12.5_Co_10_Ta_3_). The glassy alloys had better ∆T*_x_*, γ_m_, and γ values between the true and predicted composition, suggesting that a lower Si content is a necessity. Because the gas atomization process requires a metallic liquid with good fluidity that can be impinged on by high-pressure inert gas jets into fine metal droplets, the optimal composition is Ti_42_Zr_35_Si_5_Co_15_Ta_3_.

**Figure 3:**
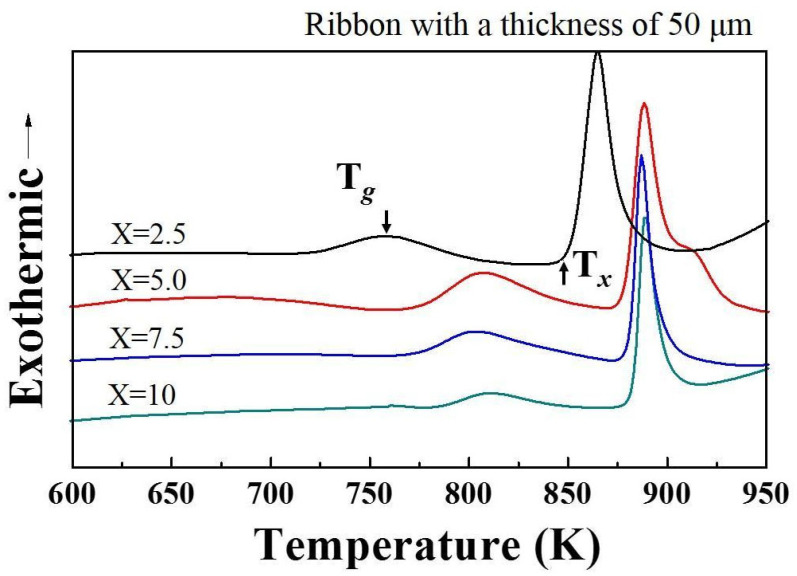
DSC curves for Ti_42_Zr_35_Si_5_Co_15-x_Sn_x_Ta_3_ (*x* = 2.5–10 in at %) glassy alloy ribbons, respectively.

The alloy composition was further fine-tuned through the substitution of Co with Sn in Ti_42_Zr_35_Si_5_Co_15-x_Sn_x_Ta_3_. Using Egami’s atomic size rule, we selected Sn as the alloying element for the following reasons. (1) The difference in atomic size between Sn and the constituent elements is large; the atomic radius of Sn is larger than the radii of Ti, Zr, Si, and Co by 1.2–30%. (2) Sn and Si belong to the same family in the periodic table, and the number of valence electrons of Sn is the same as that of Si. (3) Sn is a biofriendly element. Figure 3 illustrates the DSC curve of the Ti_42_Zr_35_Si_5_Co_15-x_Sn_x_Ta_3_ melt-spun alloy ribbons. The thermal parameters of T*_g_*, T*_x_*, ∆T*_x_*, T*_l_*, γ, and γ_m_ are summarized in Table 1. According to the results of DSC analysis, the highest ∆T*_x_* value (81 K) and a relatively high γ (0.427) and γ_m_ (0.763) occurred in the Ti_42_Zr_35_Si_5_Co_12.5_Sn_2.5_Ta_3_, which would allow the alloy to retain a high GFA.

### 3.2. Gas Atomization Process

**Figure 4:**
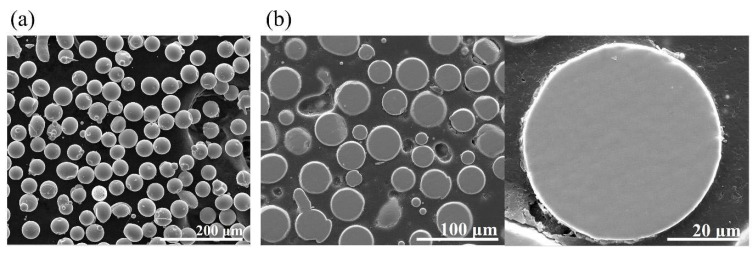
(**a**) Morphology of atomized alloy powders observed using SEM. (**b**) Cross-sectional metallography of the atomized Ti_42_Zr_35_Si_5_Co_12.5_Sn_2.5_Ta_3_ alloy powder.

**Figure 5:**
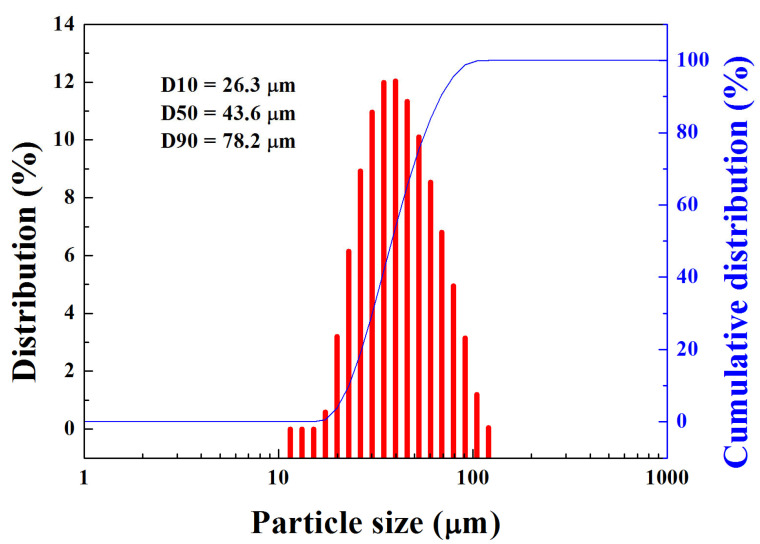
Plots of particle size distribution for as-atomized Ti_42_Zr_35_Si_5_Co_12.5_Sn_2.5_Ta_3_ powders.

**Figure 6:**
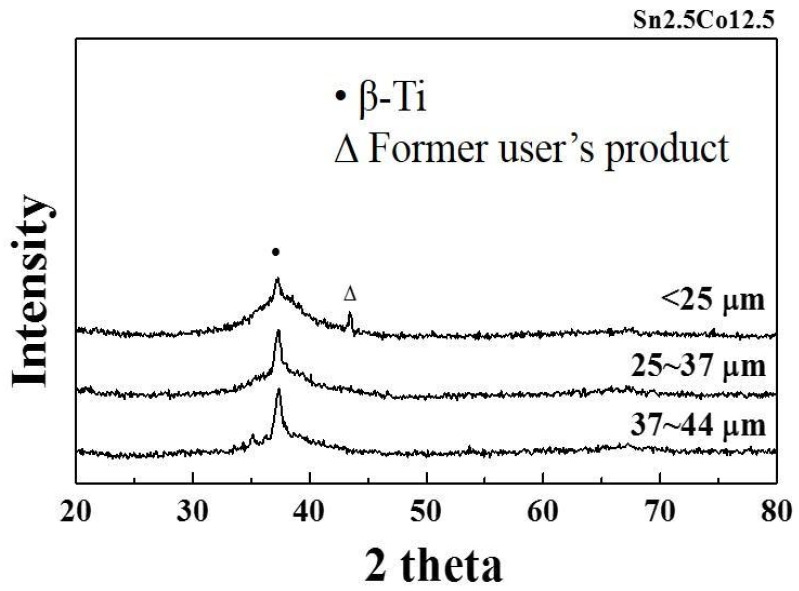
XRD patterns of atomized alloy powders for different particle size ranges.

**Figure 7:**
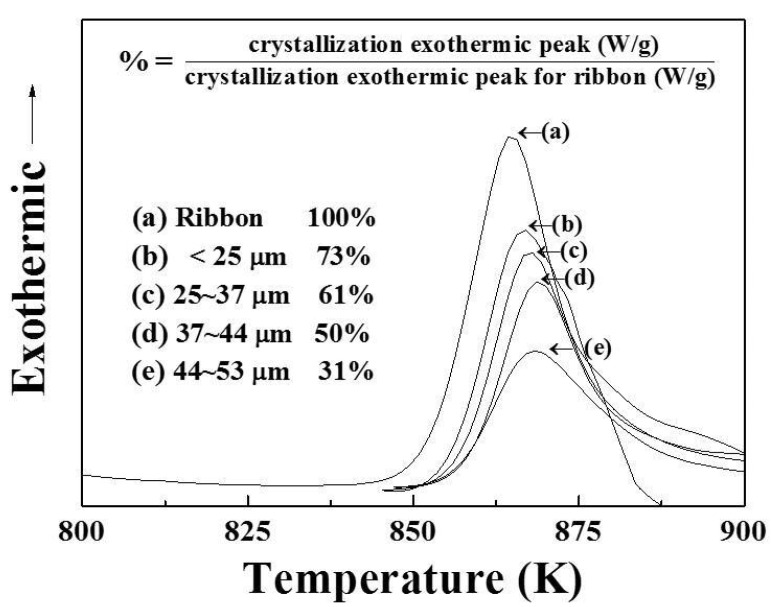
DSC curves of gas-atomized powders with different sizes and ribbons.

A master ingot with a nominal composition of Ti_42_Zr_35_Si_5_Co_12.5_Sn_2.5_Ta_3_ (in at. %) was melted in an induction vacuum furnace. High-pressure inert gas jets were impinged into fine metal droplets. The droplets settled by gravity or cyclonic separation at the bottom of the chamber to form the powder. The morphology of atomized powders, displayed in Figure 4, presents a typical spherical shape and smooth surface for the majority of powders. Only a few powders had satellite particles or an elongated shape. A cross-sectional metallography of the atomized powders clearly showed the homogenous and featureless microstructure of the solid particles. The particle size distribution of the powders is displayed in Figure 5. In addition, the yield rate for the resulting powders with a size of 15–45 μm was more than 50%. It represented the gas atomization production conditions as being finally mature and showed the fact that they would soon enter the stage of mass production. Based on systematic XRD characterization, as presented in Figure 6, Ti_42_Zr_35_Si_5_Co_12.5_Sn_2.5_Ta_3_ exhibited a dominant amorphous phase with a nanocrystalline peak, especially in powders smaller than 25 μm. The DSC analyses were conducted on gas-atomized Ti_42_Zr_35_Si_5_Sn_2.5_Co_12.5_Ta_3_ powders of different powder size ranges (<25, 25–37, 37–44, and 44–53 μm) compared with the fully amorphous ribbons prepared by melt spinning, as indicated in Figure 7. All specimens revealed characteristics of fully or partially amorphous alloys, with T*_g_* and T*_x_* and an exothermal hump over the supercooled viscous regime. We could estimate the fraction of amorphous phase from the crystallization’s exothermic peak of energy by comparing the fully amorphous state (100%) of the melt-spun ribbons. The volume fractions of the amorphous phase in the atomized Ti_42_Zr_35_Si_5_Sn_2.5_Co_12.5_Ta_3_ powders were 73% (<25 μm), 61% (25–37 μm), 50% (37–44 μm), and 31% (44–53 μm), respectively. The cooling rate of the gas atomization process was estimated to be approximately 10^2^ K/s, and the cooling rate of a typical SLM process was estimated to be greater than 10^3^ K/s [5]. Therefore, the current atomized powders can be expected to contain a higher degree of amorphous-phase volume fraction after laser remelting.

### 3.3. Selective Laser Melting

**Figure 8:**
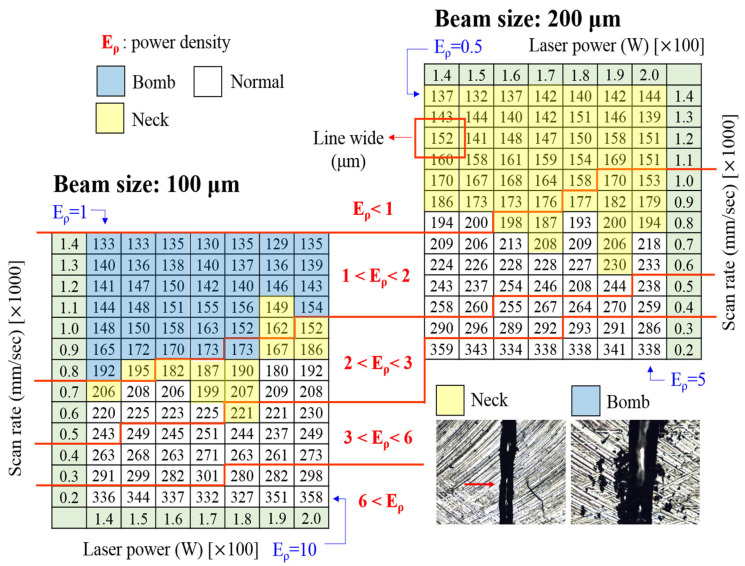
OM images of alloy lines formed after laser remelting at different energy densities for Ti_42_Zr_35_Si_5_Sn_2.5_Co_12.5_Ta_3_ (color online).

**Figure 9:**
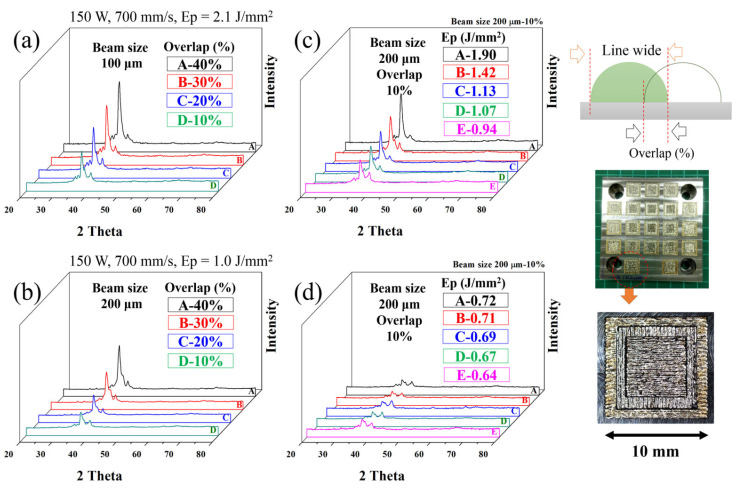
XRD pattern of SLM-plane-type samples at (**a**) beam size 100 μm, 150 W, 700 mm/s (Eρ = 2.1 J/mm^2^) with 10–40% overlaps, (**b**) beam size 200 μm, 150 W, 700 mm/s with 10–40% overlaps, (**c**) Eρ = 0.94–1.90 J/mm^2^ with 10% overlap, and (**d**) Eρ = 0.64–0.72 J/mm^2^ with 10% overlap.

**Figure 10:**
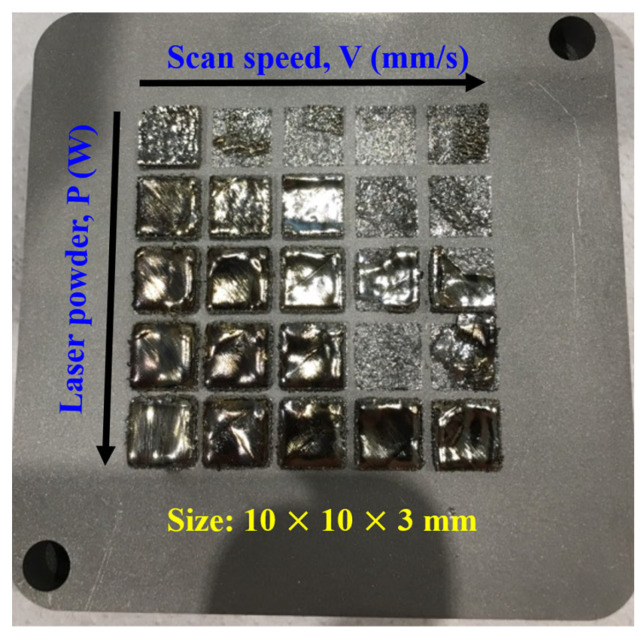
Top-view photographs of SLM volume samples.

**Figure 11:**
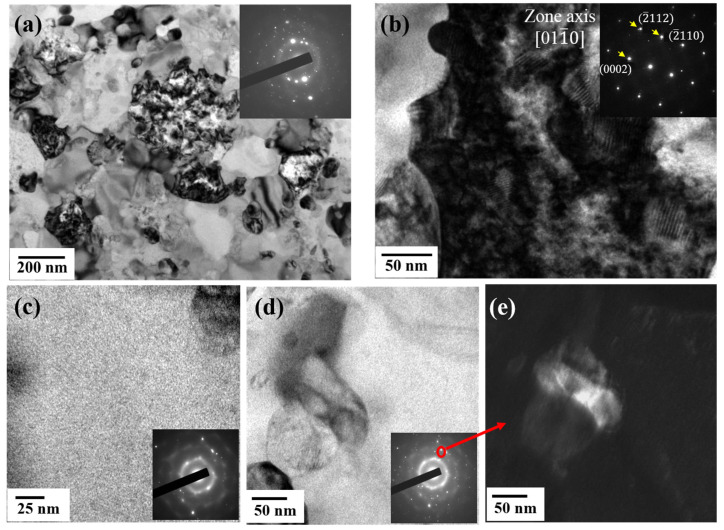
TEM image of Ti_42_Zr_35_Si_5_Co_12.5_Sn_2.5_Ta_3_ bulk metallic glass composite at scan rate of 600 mm/s, power of 120 W and overlap of 10%: (**a**) bright-field TEM image with inserted selected area diffraction (SAD) pattern for hybrid of crystalline and amorphous phase. (**b**) High-resolution image magnified from (**a**); the image illustrates that the crystalline phase has an hcp-α-Ti-type structure. (**c**) Bright-field image and SAD of amorphous phase. (**d**,**e**) Bright- and dark-field image of crystalline particle embedded in amorphous matrix.

For the analysis, the formation and microstructure were made with different laser energy densities (in J/mm^2^) Eρ = P/VD, where P is the laser power (in W), V is the scan rate (in mm/s), and D is the beam size (in μm). The gas-atomized powders with a particle size between 15 and 45 µm were examined using laser remelting for the line, plan, and volume. Figure 8 illustrates the appearance of a line formation with different laser parameters. Alloy powder easily formed an integral and continuous alloy line at the scan rate of <800 mm/s. At such a high scan speed, the powder was unable to receive sufficient energy during SLM processing, leading to incomplete melting and poor interparticle bonding. The XRD patterns for “plane” samples fabricated using different laser energy densities with a 10–40% overlap are displayed in Figure 9, with overlap occurring where an XRD pattern covered another by the crossing of its line-width edge. The crystallization rate was noticeably proportional to the overlap (Figure 9a,b) and laser energy density (Figure 9c,d), suggesting that a lower laser energy density is necessary. The energy in the laser beam heat-affected zone (HAZ) ought to be lower than that within and between the layers of the crystallization barrier for MG but must still have suitable energy levels to connect neighboring powders in order to form continuous products, as illustrated in Figure 10. This suggests that the optimal laser parameters are a scan rate of 600 mm/s, a power of 120 W, and an overlap of 10%. Figure 11 displays the bright- and dark-field TEM image of the as-built Ti_42_Zr_35_Si_5_Co_12.5_Sn_2.5_Ta_3_ bulk metallic glass. The selected area’s diffraction patterns corresponding to the zone axis (01$\bar{1}$0) of the hcp-α-Ti-type and amorphous structure are displayed in Figure 11b,c, respectively. This pattern indicates that the as-built sample by SLM is in a composite state. When comparing the cooling rate of the gas atomization process (10^2^ K/s) with the cooling rate of the SLM process (>10^3^ K/s) [5], we expected to observe a higher amorphous-phase volume fraction in the SLM block than in the atomized powder (the volume fraction of the atomized powders was 50–73%). However, the volume fraction of the amorphous phase in the SLM block was only 40–50%, according to the TEM image analysis (Figure 11a). This suggests that the partial crystallization of the SLM block was induced by the HAZ or the repeated thermal cycle during the laser remelting of each new layer.

The SLM process includes the remelting and solidification of the metallic powders. When the cooling rate is fast enough to suppress crystallization behavior, an MG can be formed. The high GFA of the alloy composition contributes greatly to this. Additionally, inattention to the HAZ may result in crystallization because of the limited thermal stability of the amorphous alloy’s crystallization temperature. This problem may be overcome by decreasing the substrate temperature and reducing the HAZ’s adverse effects or by changing the laser scan strategy to avoid heat clustering as much as possible.

## 4. Conclusions

The Ti_42_Zr_35_Si_5_Co_12.5_Sn_2.5_Ta_3_ BMGC has been successfully fabricated by SLM. The influence of the processing parameters on the microstructure of the fabricated ribbon, powder, and BMGCs was systematically investigated. This study makes the following contributions.

1. A new biocompatible Ti-based MG, Ti_42_Zr_35_Si_5_Co_12.5_Sn_2.5_Ta_3_, with high GFA was synthesized. The glassy alloy exhibited a relatively high thermal stability with a supercooled liquid region (∆T*_x_*) above 81 K. The T*_g_*, T*_x_*, T*_l_*, γ, and γ_m_ were 761 K, 842 K, 1210 K, 0.427, and 0.763, respectively.
2. The morphology of the atomized powders presented a typical spherical shape and smooth surface for the majority of the powder. When subject to systematic XRD characterization, the powder exhibited a dominant amorphous phase with some nanocrystalline peaks. The volume fractions of the amorphous phase in the atomized Ti_42_Zr_35_Si_5_Sn_2.5_Co_12.5_Ta_3_ powders characterized by DSC were 73% (<25 μm), 61% (25–37 μm), 50% (37–44 μm), and 31% (44–53 μm), respectively.
3. The gas-atomized powders with a particle size between 15 and 45 µm were examined by laser remelting for the line, plan, and volume. Achieving a lower laser energy density was a top priority because the energy in the laser beam HAZ ought to be lower than the crystallization barrier for MG within and between layers but must still have a suitable energy level to connect neighboring powders in order to form continuous products. The optimal laser parameters are a scan rate of 600 mm/s, a power of 120 W, and an overlap of 10%.
4. The microstructure of biocompatible Ti-based bulk metallic glass composites for additive manufacturing consists of an hcp-α-Ti-type and amorphous structure.

SLM technology offers an opportunity to manufacture a range of new products. Because Ti-based MG exhibits good biocompatible properties, the complex, customizable, and fast-yield products of Ti-based BMGC by SLM are promising candidates for biomedical implants.

## Figures and Tables

**Table 1:** Thermal properties of as-quenched base Ti_42_Zr_40_Ta_3_Si_15_ and other newly designed alloys with various combinations of Sn and Co additions.

Alloys	T*_g_*(K)	T*_x_*(K)	∆T*_x_*(K)	T*_l_*(K)	γ	γ_m_
Base Ti_42_Zr_40_Si_15_Ta_3_	799	898	99	1728	0.355	0.577
Ti_42_Zr_35_Si_5_Co_15_Ta_3_	745	817	72	1201	0.420	0.740
Ti_42_Zr_35_Si_5_Co_12.5_Sn_2.5_Ta_3_	761	842	81	1210	0.427	0.763
Ti_42_Zr_35_Si_5_Co_10_Sn_5_Ta_3_	809	873	64	1212	0.432	0.773
Ti_42_Zr_35_Si_5_Co_7.5_Sn_7.5_Ta_3_	803	874	71	1198	0.437	0.789
Ti_42_Zr_35_Si_5_Co_2.5_Sn_12.5_Ta_3_	815	874	59	1200	0.434	0.778

## Data Availability

Data is contained within the article.
